# Outbreak-associated Novel Duck Reovirus, China, 2011

**DOI:** 10.3201/eid1807.120190

**Published:** 2012-07

**Authors:** Zongyan Chen, Yinqi Zhu, Chuanfeng Li, Guangqing Liu

**Affiliations:** Shanghai Veterinary Research Institute–Chinese Academy of Agricultural Sciences, Shanghai, People’s Republic of China

**Keywords:** ducks, reovirus, avian, orthoreoviruses, outbreaks, viruses, People’s Republic of China, Pekin ducks, *Anas platyrhynchos*

**To the Editor:** In 2011, an unidentified disease in Pekin ducks (*Anas platyrhynchos*) was reported in People’s Republic of China. The infection caused death in 40% of ducks of various age and 35%–40% mortality in different flocks. Clinical signs included unstable gait, weakness in legs, and diarrhea. At necropsy, large necrotic foci were observed in the spleens. All classical endemic and emerging viruses, such as duck enteritis virus, duck hepatitis virus, duck flavivirus, duck parvovirus, and avian influenza virus, could be excluded as the causative agent by PCR and serologic methods. To identify the cause of the disease, we tested tissue from affected ducks and subsequently isolated a novel duck-pathogenic orthoreovirus from the livers of affected ducks.

Avian orthoreoviruses (ARVs) belong to the family *Reoviridae*, genus *Orthoreovirus* (*1*). The virions are nonenveloped, with icosahedral symmetry and a double capsid containing 10 double-stranded RNA segments that can be separated by polyacrylamide gel electrophoresis into 3 size classes: large (L1–L3), medium (M1–M3), and small (S1–S4) (*2**,**3*). ARVs cause a range of diseases in chicken, including viral arthritis/tenosynovitis, and are associated with respiratory disease, enteric disease, inclusion body hepatitis, hydropericardium, runting stunting syndrome, malabsorption syndrome, and sudden death. ARVs also have been isolated from the Muscovy duck (*Cairina moschata*). Muscovy duck reovirus infection caused illness in 30% and death in 20% of ducks on poultry farms in Israel (*4*). In China, reovirus infection has been reported in Muscovy ducklings, with a resulting death rate of 10%–30% since 1997 (*5*). The isolated reovirus was highly pathogenic to 1-day-old Muscovy ducklings by experimental infection. However, the Muscovy duck reovirus isolate was nonpathogenic for Pekin ducks when inoculated subcutaneously (*4*).

Since 2007, three isolates of orthoreovirus were confirmed in Pekin ducks from several duck farms in China. However, experiment infection with the isolates did not cause death (*6*). In 2011, farmers and veterinarians in China reported to the Animal Health Services and National Research Institutes an unidentified disease in ducks that spread rapidly around the county. We conducted further investigation to identify the causative agent of this disease. The diseased ducks showed depression and leg weakness. Large necrotic foci were observed in the spleens of the dead ducks. Histopathologic examination showed necrotic foci and granulomas in the spleen. Focal hepatic necrosis and proliferation of bile ducts were seen in the liver. Virus isolation from liver homogenate was conducted in duck embryo fibroblast cultures. At 48 hours after infection, a strong cytopathic effect was observed, including syncytium formation. All duck embryos experimentally infected with the isolate died within 48–72 hours after infection. The dead embryos showed swollen livers with petechial hemorrhages. Spherical, spiked virus particles, consistent with those of members of the family *Reoviridae*, were observed by electron microscopy. As reported (*7*), the diameter of the particles was ≈85 nm (Figure A1). The RNA extracted from DRV-infected duck embryo fibroblast cultures showed 10 dsRNA segments in 3 size classes (L1–3, M1–3, and S1–4) on polyacrylamide gel electrophoresis. The isolate was designated as novel duck reovirus, DRV-TH11. The pathogenicity of DRV-TH11 was tested by infecting 10-day-old Pekin ducks subcutaneously at a dose of 4 × 10^4.5^ 50% tissue culture infective dose. Experimental infection caused death on day 3 after infection. The clinical signs and histopathologic examination show the same features as the naturally infected ducks.

**Figure:**
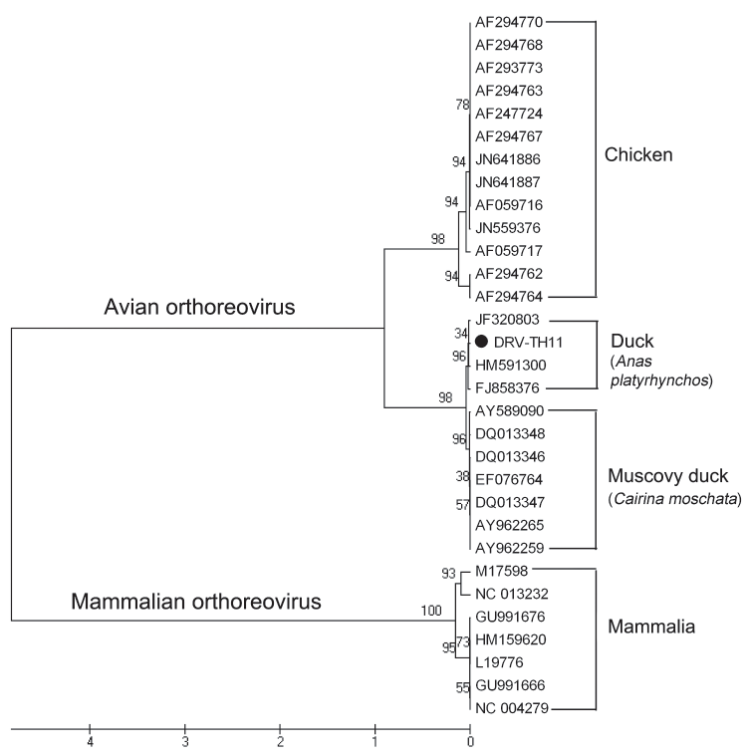
Phylogenetic relationship between DRV-TH11 isolate and orthoreovirus of the avian orthoreovirus (ARV) and mammalian orthoreovirus (MRV). ARV includes chicken reovirus, Muscovy duck reovirus, and Pekin duck reovirus. GenBank accession numbers of the sequences in the analysis are indicated in the tree. The neighbor-joining tree is based on the complete sequence of s2 gene (1,251 nt). Numbers at nodes represent the percentage of 1,000 bootstrap replicates (values <50 are not shown). Scale bar indicates a branch length corresponding to 100 character-state changes.

For phylogenetic analyses, the S2 gene was amplified by reverse transcription PCR with avian reovirus–specific primers. The complete sequence of the S2 gene (GenBank accession no. JQ664689) was aligned with 30 published orthoreovirus sequences, including data on all 3 newly obtained sequences from Pekin duck reovirus in China in 2008 and 2011. Phylogenetic relationship was assessed by using the neighbor-joining method based on a Tamura 3-parameter model and bootstrap analysis (1,000 replicates) as implemented in MEGA5 (*8*). The phylogenetic tree shows that the complete sequence of S2 gene is distinct but clusters closely with sequences from all 3 Pekin duck isolates within the ARVs serogroup, which suggests that the novel virus is an ARV-like virus within the genus *Orthoreovirus* (Figure).

In summary, we isolated a novel duck-pathogenic orthoreovirus from the liver of affected Pekin ducks. The regression test in its natural host animal showed that the newly isolated virus caused their deaths. This finding highlights the need to prevent and control this highly transmissible infectious agent. Further study is needed to determine what role the newly isolated DRV played in the 2011 outbreaks on many of the duck farms in China.

## Figures and Tables

**Figure A1:**
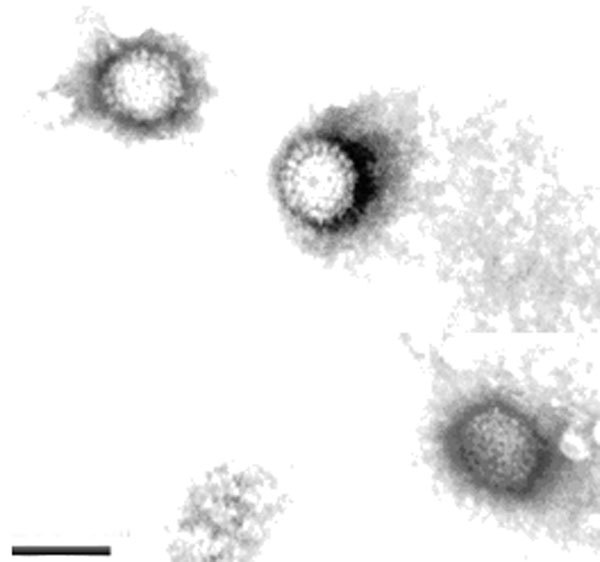
Electron micrographs of purified DRV-TH11 isolate particles showing the features of orthoreovirus. Original magnification ×100,000. Scale bar = 100 nm.
